# Automated whole-slide images assessment of immune infiltration in resected non-small-cell lung cancer: towards better risk-stratification

**DOI:** 10.1186/s12967-022-03458-9

**Published:** 2022-06-07

**Authors:** Huan Lin, Xipeng Pan, Zhengyun Feng, Lixu Yan, Junjie Hua, Yanting Liang, Chu Han, Zeyan Xu, Yumeng Wang, Lin Wu, Yanfen Cui, Xiaomei Huang, Zhenwei Shi, Xin Chen, Xiaobo Chen, Qingling Zhang, Changhong Liang, Ke Zhao, Zhenhui Li, Zaiyi Liu

**Affiliations:** 1 School of Medicine, South China University of Technology, Guangzhou, 510006 China; 2 Department of Radiology, Guangdong Provincial People’s Hospital, Guangdong Academy of Medical Sciences, Guangzhou, 510080 China; 3 Guangdong Provincial Key Laboratory of Artificial Intelligence in Medical Image Analysis and Application, Guangdong Provincial People’s Hospital, Guangdong Academy of Medical Sciences, Guangzhou, 510080 China; 4 Guangdong Cardiovascular Institute, Guangzhou, 510080 China; 5 School of Computer Science and Information Security, Guilin University of Electronic Technology, Guilin, 541004 China; 6 Department of Pathology, Guangdong Provincial People’s Hospital, Guangdong Academy of Medical Sciences, Guangzhou, 510080 China; 7 Department of Epidemiology and Health Statistics, Hunan Provincial Key Laboratory of Clinical Epidemiology, Xiangya School of Public Health, Central South University, Changsha, 410078 China; 8 Department of Pathology, The Third Affiliated Hospital of Kunming Medical University, Yunnan Cancer Hospital, Yunnan Cancer Center, Kunming, 650118 China; 9 The Second School of Clinical Medicine, Southern Medical University, Guangzhou, 510515 China; 10 Department of Radiology, Guangzhou First People’s Hospital, Guangzhou, 510180 China; 11 First Department of Thoracic Surgery, The Third Affiliated Hospital of Kunming Medical University, Yunnan Cancer Hospital, Yunnan Cancer Center, Kunming, 650118 China; 12 Department of Radiology, The Third Affiliated Hospital of Kunming Medical University, Yunnan Cancer Hospital, Yunnan Cancer Center, Kunming, 650118 China

**Keywords:** Non-small-cell lung cancer (NSCLC), Whole-slide image, Immunohistochemistry (IHC), Tumour immune microenvironment, Prognosis prediction

## Abstract

**Background:**

High immune infiltration is associated with favourable prognosis in patients with non-small-cell lung cancer (NSCLC), but an automated workflow for characterizing immune infiltration, with high validity and reliability, remains to be developed.

**Methods:**

We performed a multicentre retrospective study of patients with completely resected NSCLC. We developed an image analysis workflow for automatically evaluating the density of CD3^+^ and CD8^+^ T-cells in the tumour regions on immunohistochemistry (IHC)-stained whole-slide images (WSIs), and proposed an immune scoring system “I-score” based on the automated assessed cell density.

**Results:**

A discovery cohort (n = 145) and a validation cohort (n = 180) were used to assess the prognostic value of the I-score for disease-free survival (DFS). The I-score (two-category) was an independent prognostic factor after adjusting for other clinicopathologic factors. Compared with a low I-score (two-category), a high I-score was associated with significantly superior DFS in the discovery cohort (adjusted hazard ratio [HR], 0.54; 95% confidence interval [CI] 0.33–0.86; *P* = 0.010) and validation cohort (adjusted HR, 0.57; 95% CI 0.36–0.92; *P* = 0.022). The I-score improved the prognostic stratification when integrating it into the Cox proportional hazard regression models with other risk factors (discovery cohort, C-index 0.742 vs. 0.728; validation cohort, C-index 0.695 vs. 0.685).

**Conclusion:**

This automated workflow and immune scoring system would advance the clinical application of immune microenvironment evaluation and support the clinical decision making for patients with resected NSCLC.

**Supplementary Information:**

The online version contains supplementary material available at 10.1186/s12967-022-03458-9.

## Background

Lung cancer is the leading cause of cancer-related deaths worldwide [1], and non-small-cell lung cancer (NSCLC) is the most common subtype of lung cancer, which comprises 85% of total diagnoses [2]. Surgery is the recommended treatment for resectable NSCLC [3], whereas 30–55% of patients develop recurrence and die despite the resection [4]. Precise risk assessment is crucial for developing individualized treatment strategies. The American Joint Committee on Cancer (AJCC) tumour-node-metastasis (TNM) staging system [5] is widely used for risk stratification, but patients prognosis varies within each stage due to biological heterogeneity [2]. Prediction models combining the TNM stage and clinicopathologic prognostic factors (e.g. histologic type, and treatment-related factors) have improved the clinical validity of risk stratification, but the predictive performance is unsatisfactory [6–8]. A novel prognostic biomarker that characterizes the biological behaviour may improve the validity of risk stratification in NSCLC.

Recent tumour biological studies have implied that the interaction between the tumours and microenvironment is associated with tumour development, invasion, metastasis, and outcome [9, 10]. Tumour-infiltrating lymphocytes (TILs) within the microenvironment has been reported to be the prognostic factor of resected NSCLC [11], among which T-cells (CD3^+^), especially cytotoxic T-cells (CD8^+^), play important roles in antitumour immunity [12, 13]. In recent years, many studies have attempted to characterize the in situ immune infiltration based on the density of various T-cells subsets (e.g. CD8^+^, CD3^+^, CD4^+^, FOX-P3^+^, CD45RO^+^, etc.) [10, 14]. However, a generally accepted immune scoring system for NSCLC is still unavailable since there is no consensus regarding the selection of T-cells subsets and the cell quantification approaches [13, 14].

Immunohistochemistry (IHC) on tissue sections is a simple and reliable method to identify CD3^+^ and CD8^+^ T-cells. The conventional method for quantifying positive cells is through manual counting performed by pathologists, which is time-consuming with poor reproducibility. There have been prior attempts at automated histopathological analysis based on NSCLC tissue microarrays (TMA), such as evaluating the density and spatial arrangement of TILs [15], and quantifying the different subsets of T-cells [16, 17]. However, the selection bias of TMAs may lead to high inter-observer variability [18]. In comparison, computer-aided analyses based on digitalized whole-slide images (WSIs) evaluate the whole tissue sections without subjective selection of regions for analysis, which improve reproducibility across users, and the spatial heterogeneity within the tumour microenvironment could be better characterized [19]. Automated workflows for evaluating the immune infiltration on IHC-stained WSIs are expected to improve the validity and reliability of NSCLC risk stratification [20, 21], but such an algorithm remains to be developed.

This study aimed to achieve the following objectives using 2 retrospective cohorts of patients with resected NSCLC. Firstly, we developed an automated workflow for evaluating the density of CD3^+^ and CD8^+^ cells in the tumour regions on IHC-stained WSIs. Secondly, we proposed an immune scoring system based on the automated assessed cell density. We hypothesised that the integration of this immune scoring system into clinicopathological risk factors would improve the prognostic stratification in resected NSCLC.

## Methods

### Patients cohorts

**Fig. 1:**
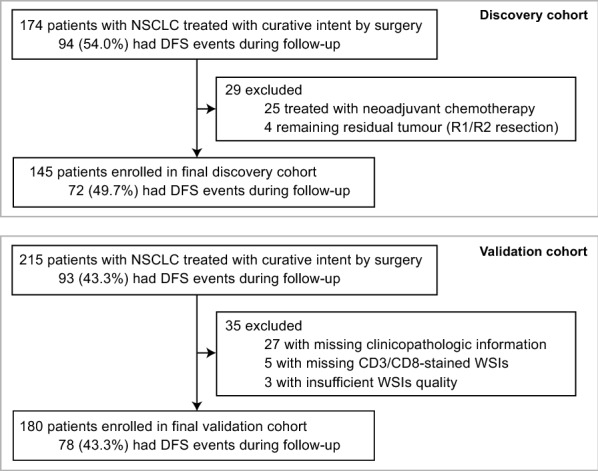
Discovery cohort and validation cohort enrolment, exclusions, and incidence of DFS events during follow up. *NSCLC* non-small-cell lung cancer, *DFS* disease-free survival, *WSI* whole slide image

This retrospective study was conducted using two independent cohorts of patients: a discovery cohort (Guangdong Provincial People’s Hospital) and a validation cohort (Yunnan Cancer Hospital) (Fig. 1). The Institutional Ethics Committees at Guangdong Provincial People’s Hospital (approval number: KY-Z-2021-030-02) and Yunnan Cancer Hospital (approval number: KY2020139) approved the use of WSIs of IHC-stained tissue sections, and informed consent was waived because only retrospective imaging analysis was performed. Consecutive patients with NSCLC who were treated with curative intent by surgery between 2007 and 2015 were enrolled. The patients that were treated with neoadjuvant therapy, remained residual tumour (R1/R2 resection), or died within 30 days after surgery were excluded. The endpoint of interest for this study was disease-free survival (DFS), which was defined as the time from surgery to the first recurrence, or death. Patients underwent followed-up (contrast-enhanced chest computed tomography or phone interview) once every 6 months for the first 2 years, and then annually. The duration of follow-up was calculated from the time of surgery until the occurrence of the event or the last follow-up, and information about the survival status was documented. Baseline and clinicopathologic characteristics, including age at surgery, sex, smoking history, pT stage, pN stage, TNM stage, tumour location, histologic type, differentiation grade, type of surgery, and adjuvant chemotherapy were collected from the medical records. Patients with any missing clinicopathologic information or WSIs for analysis were excluded, and no imputation of missing values was performed. The TNM stage was manually reviewed to ensure that it corresponded to the ﻿American Joint Committee on Cancer (AJCC) staging system (8th edition, 2017) [5]. Adjuvant chemotherapy protocols were standardized according to National Comprehensive Cancer Network (NCCN) guidelines [3].

### IHC-stained sections digitalization

**Fig. 2:**
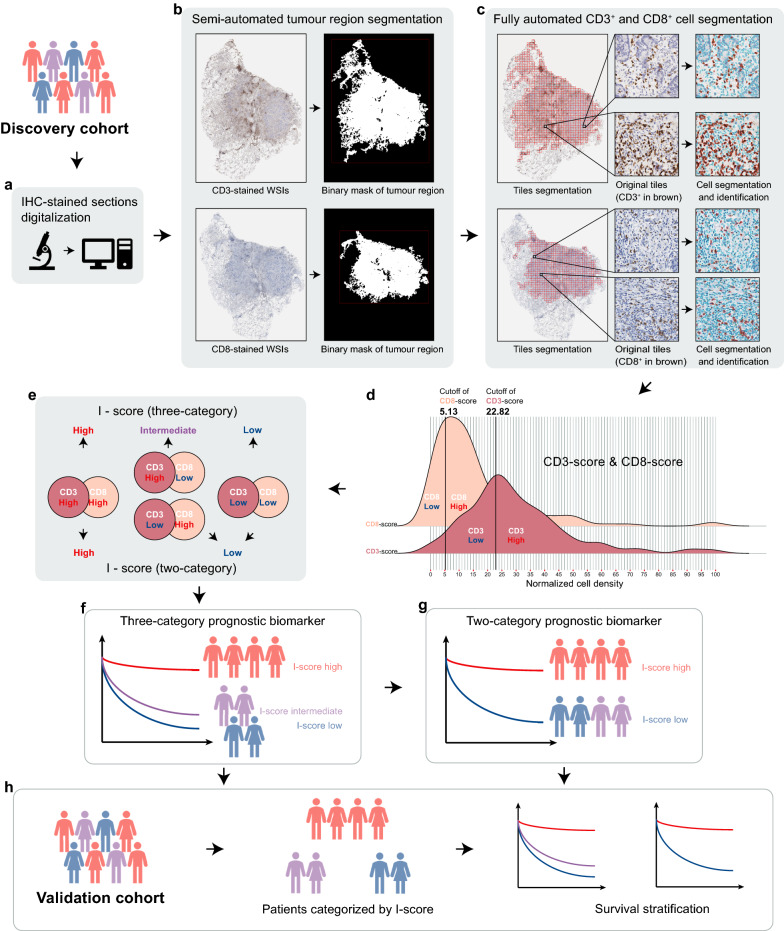
Overall workflow of this study. **a** Two adjacent sections stained with CD3 and CD8 are digitalized by using the whole-slide scanner. **b** By using a semi-automated image analysis algorithm, a binary mask of segmented tumour region is created for each WSI. **c** The CD3^+^ and CD8^+^ T-cells in the tumour region are segmented and identified by using a fully-automated algorithm. **d** The CD3-score and CD8-score (normalized CD3^+^ and CD8^+^ cell density, ranging from 0 to 100) are classified into low and high based on the cutoffs determined by maximally selected rank statistics, respectively. **e**, **f**, **g** A three-category I-score and a two-category I-score are established by integrating the classifications of CD3-score and CD8-score based on the discovery dataset. **h** The validation cohort is used to assess the prognostic value of the I-score

The surgical specimens of NSCLC were fixed by formalin and embedded in paraffin. Tumour sections were selected from the tissue blocks by an experienced pathologist from each hospital (LXY and LW) who were blinded to clinical information. Ensured that the selected tissue sections were complete and avoided large necrotic areas. Two adjacent sections were stained with anti-CD3 and anti-CD8. Full details of the IHC staining was presented in Additional file 1: Note S1. The IHC-stained sections were digitalized by using the whole-slide scanner (Leica, Aperio-AT2, USA) at 40 × magnification with a resolution of 0.252 μm per pixel (Fig. 2a). We performed quality control manually by excluding artefacts, blurry images, and light- or over- stained tissues (Fig. 1).

### Tumour region segmentation

Segmentation of the tumour region was a semi-automated interactive process. WSIs were first downsampled by a factor of 16, and then all three colour channels of Red–Green–Blue (RGB) were extracted. By converting the downsampled RGB images into Hue-Saturation-Value (HSV) colourspace, the H and S channels were then extracted. Ostu’s segmentation algorithm [22] with automatic threshold was used to determine and segment the boundaries of tumour regions, removing adjacent normal tissue, blank area, and background. All segmentation results were visually checked by 2 pathologists (LXY and LW), and if required, the algorithm parameters were fine-tuned to precisely determine the boundaries of the tumour region (ZYF). A binary mask of segmented tumour region was created for each WSI, for later processing (Fig. 2b).

### CD3^+^ and CD8^+^ T cell segmentation and quantification

Segmentation and quantification of CD3^+^ and CD8^+^ cells were fully-automated processes. The tumour regions were first tiled into non-overlapping patches of 1024 × 1024 pixels (40 × magnification), ensuring that the tumour area on each tile occupied no less than 50% of the entire tile area. The tile-level CD3^+^/CD8^+^ cell segmentation pipeline included: dye channel separation, background/blank areas and dust macules removal, Bernsen-based local threshold segmentation, and watershed segmentation of adhesive cells. Firstly, the Hematoxylin and DAB channels of IHC-stained tiles were extracted using a colour deconvolution algorithm. Secondly, the super-pixel segmentation method and k-means clustering algorithm were employed to remove the background/blank areas. The super-pixel segmentation method divided the image into irregular super-pixel blocks, and the k-means clustering algorithm was used to distinguish the background/blank area. Dust macules, which were specific to the lung tissues, were filtered out using a fixed threshold. Thirdly, morphological features of cells were used for preliminary image segmentation, and Bernsen-based local threshold segmentation was employed to further improve the segmentation accuracy. Lastly, adherent cells segmentation was carried out based on the watershed algorithm. The results of cell segmentation and identification were overlaid on tiles (Fig. 2c). The density of CD3^+^/CD8^+^ cells was calculated as the counting of CD3^+^/CD8^+^ cells per unit of tissue surface area (mm^2^, including only tumour area, excluding spaces and background).

### Comparison of automated and manual counting of positive cells

To evaluate the agreement between manual counting and automated counting of positive cells, 60 tiles from the WSIs in the discovery cohort (30 CD3-tiles and 30 CD8-tiles) and 60 tiles from the WSIs in the validation cohort (30 CD3-tiles and 30 CD8-tiles) were randomly selected. The gold standard of positive cell identification was determined by a lung pathologist (LXY) who was blind to the result of cell segmentation. The manual annotation was performed using QuPath 0.3.2 (https://qupath.github.io/).

Furthermore, the performance of our algorithm was compared to the QuPath built-in algorithm. Jointly considering of the size of lymphocytes (5–10 μm in diameter) and segmentation performance, the minimum cell area threshold was at 100, 150, or 200 pixel^2^, respectively, and other parameters were maintained at default in QuPath software.

### I-score establishment

To facilitate the use of CD3^+^ and CD8^+^ cell density, they were normalized into CD3-score and CD8-score respectively, ranging from 0 to 100. The CD3-score and CD8-score were classified into low and high based on the cutoffs determined by maximally selected rank statistics method (Fig. 2d, Additional file 1: Figure S2, Additional file 1: Figure S3). We developed a three-category and a two-category immune scoring system “I-score” by integrating the classifications of CD3-score and CD8-score based on the discovery dataset (Fig. 2e). The three-category I-score was defined as high when both the CD3-score and CD8-score were classified as high; defined as intermediate when one of the CD3-score and CD8-score was classified as high; and defined as low when both the CD3-score and CD8-score were classified as low. The two-category I-score was defined as high when both the CD3-score and CD8-score were classified as high; and defined as low in other cases (combining the I-score-low and I-score-intermediate groups in the three-category scoring system).

### Statistical analysis

Continuous data with non-normal distributions were reported as median (interquartile range, IQR) and compared via Mann–Whitney U test. Categorical data were reported as count (percentage) and compared via Pearson Chi-square test. The median follow-up between the two cohorts was compared by the reverse Kaplan–Meier method. The association between I-score and TNM stage was analysed using linear-by-linear association. The agreement between manual cell counting and automated cell counting was assessed by Bland–Altman plot and intraclass correlation coefficient (ICC).

The Kaplan–Meier curves and Cox proportional hazards models were used for survival analyses. The proportional hazards assumption was tested using the Schoenfeld residuals test and log–log plots, and the assumption was not violated. The association between risk factors (I-score and clinicopathologic characteristics) and DFS were evaluated using univariable Cox models. Variables that reached statistical significance at *P* < 0.10 in the univariable analysis were candidates for the multivariable Cox models. The final model (full model) was determined using stepwise regression based on the Akaike information criterion (AIC). Model discrimination was evaluated using the integrated area under the curve [23] (iAUC, resampling with 1000 times bootstrapping) and Harrell’s concordance index (C-index) [24]. The iAUC and C-index of 1 indicated perfect concordance, and 0.5 indicated random prediction. Model calibration was evaluated by AIC, and a lower AIC indicated better calibration. The model performance was compared using the likelihood ratio test [25].

Statistical analyses were conducted using SPSS 20.0 (SPSS Inc., Chicago, IL, USA) and R 4.0.3 (R Foundation for Statistical Computing, Vienna, Austria) with packages survival, survminer, Hmisc, gbm, MASS, risksetROC, lmtest. A two-tailed *P*-value < 0.05 was considered statistically significant. The retrospective nature of this study predetermines the sample size. Hence, the maximum number of candidate risk factors was determined as 7 based on the number of events in the discovery cohort, to ensure that there were at least 10 events per candidate predictor (10 EPP rule [26]).

## Results

### Patients characteristics

Based on the inclusion and exclusion criteria, 145 patients (72 events occurred during follow-up) were enrolled in the final discovery cohort, and 180 patients (78 events occurred during follow-up) were enrolled in the final validation cohort (Fig. 1). Median (IQR) follow-up was 102.7 (89.7–115.6) months for the discovery cohort and 60.0 (57.1–62.8) months for the validation cohort. Baseline and clinicopathologic characteristics of the two cohorts are shown in Table 1. There were significant differences between the two cohorts in age at surgery, smoking history, pT stage, histologic type, differentiation grade, type of surgery, and adjuvant chemotherapy (*P* < 0.050, Table 1).

**Table 1:** Baseline and clinicopathologic characteristics of the patients with NSCLC in the discovery and validation cohorts

	Discovery cohort	Validation cohort	*P*
Age at surgery (year, median [IQR])	61.0 (54.5–67.0)	56.0 (49.0–63.0)	< 0.001^a^
< 65	95 (65.5%)	143 (79.4%)	0.005^b^
≥ 65	50 (34.5%)	37 (20.6%)	
Sex			0.997^b^
Male	83 (57.2%)	103 (57.2%)	
Female	62 (42.8%)	77 (42.8%)	
Smoking history			0.027^b^
Never	101 (69.7%)	104 (57.8%)	
Former/current	44 (30.3%)	76 (42.2%)	
pT stage			< 0.001^b^
T1	44 (30.3%)	132 (73.3%)	
T2	78 (53.8%)	33 (18.3%)	
T3	16 (11.0%)	7 (3.9%)	
T4	7 (4.8%)	8 (4.4%)	
pN stage			0.382^b^
N0	109 (75.2%)	132 (73.3%)	
N1	12 (8.3%)	23 (12.8%)	
N2	24 (16.6%)	25 (13.9%)	
TNM stage			0.815^b^
I	92 (63.4%)	114 (63.3%)	
II	21 (14.5%)	30 (16.7%)	
III	32 (22.1%)	36 (20.0%)	
Tumour location			0.051^b^
Upper/middle lobe	96 (66.2%)	100 (55.6%)	
Lower lobe	49 (33.8%)	80 (44.4%)	
Histologic type			0.001^c^
Adenocarcinoma	111 (76.6%)	143 (79.4%)	
Squamous cell carcinoma	23 (15.9%)	37 (20.6%)	
Other	11 (7.6%)	0 (0.0%)	
Differentiation grade			0.005^b^
Well-moderately differentiated (G1/G2)	107 (73.8%)	106 (58.9%)	
Poorly-undifferentiated (G3/G4)	38 (26.2%)	74 (41.1%)	
Type of surgery			0.046^b^
Lobectomy/pneumonectomy	134 (92.4%)	175 (97.2%)	
Limited resection	11 (7.6%)	5 (2.8%)	
Adjuvant chemotherapy			< 0.001^b^
No	94 (64.8%)	74 (41.1%)	
Yes	51 (35.2%)	106 (58.9%)	
Follow-up duration (month, median [95% CI])	102.7 (89.7–115.6)	60.0 (57.1–62.8)	< 0.001^d^
No. of events	72 (49.7%)	78 (43.3%)	0.256^b^

Data in parentheses are IQR, percentages or 95% confidence intervals

*NSCLC* non-small-cell lung cancer, *IQR* interquartile range, *CI* confidence interval

^a^*P*-value is determined by Mann–Whitney U test

^b^*P*-values are determined by Pearson Chi-square test

^c^*P*-value is determined by Chi-square test with continuity correction

^d^*P*-value is determined by the reverse Kaplan–Meier method

### Segmentation results and Bland–Altman analysis

**Fig. 3:**
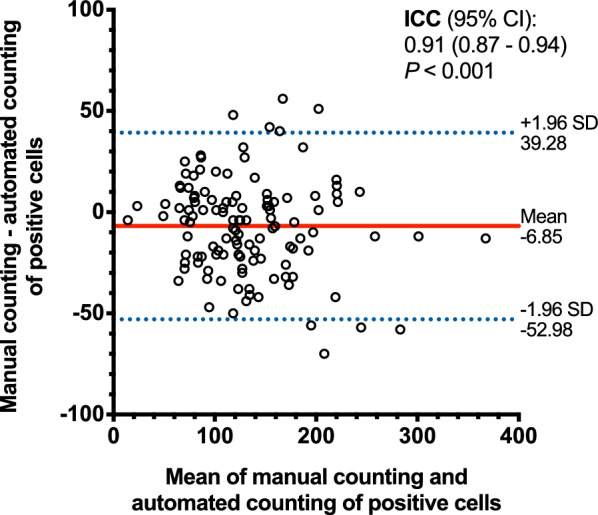
Bland–Altman plot for agreement between manual counting and automated counting using our algorithm for quantifying CD3^+^/CD8^+^ cells. The Bland–Altman analysis is performed by using 120 tiles randomly selected from the WSIs in the discovery cohort (30 CD3-tiles and 30 CD8-tiles) and the validation cohort (30 CD3-tiles and 30 CD8-tiles). The solid horizontal line in red is the mean of the difference between manual counting and automated counting of positive cells, and the dashed lines in blue are the upper/lower bounds of 95% limits of agreement (95% LoA). The intraclass correlation coefficient (ICC) is 0.91 (95% confidence interval, 0.87–0.94; *P* < 0.001), which indicates good agreement between manual counting and automated counting. *ICC* intraclass correlation coefficient. Data in parentheses are 95% confidence intervals

The results of tumour region segmentation and CD3^+^/CD8^+^ T-cells segmentation were shown in Fig. 2b and Fig. 2c. Totally 120 tiles were randomly selected from the discovery cohort and validation cohort to evaluate the agreement between manual counting and automated counting of positive cells. Our algorithm (Additional file 1: Figure S1a) showed better segmentation performance compared to the QuPath built-in algorithm (Additional file 1: Figure S1c, e, g), regardless of cell area threshold (100, 150, or 200 pixel^2^). The Bland–Altman plot showed good agreement between the manual counting and automated counting using our algorithm (ICC, 0.91; 95% confidence interval [CI], 0.87–0.94; *P* < 0.001; Fig. 3), but showed moderate agreement between the manual counting and automated counting using QuPath built-in algorithm (ICC, 0.44–0.72; Additional file 1: Figure S1d, f, h).

### Prognostic value of I-score

**Fig. 4:**
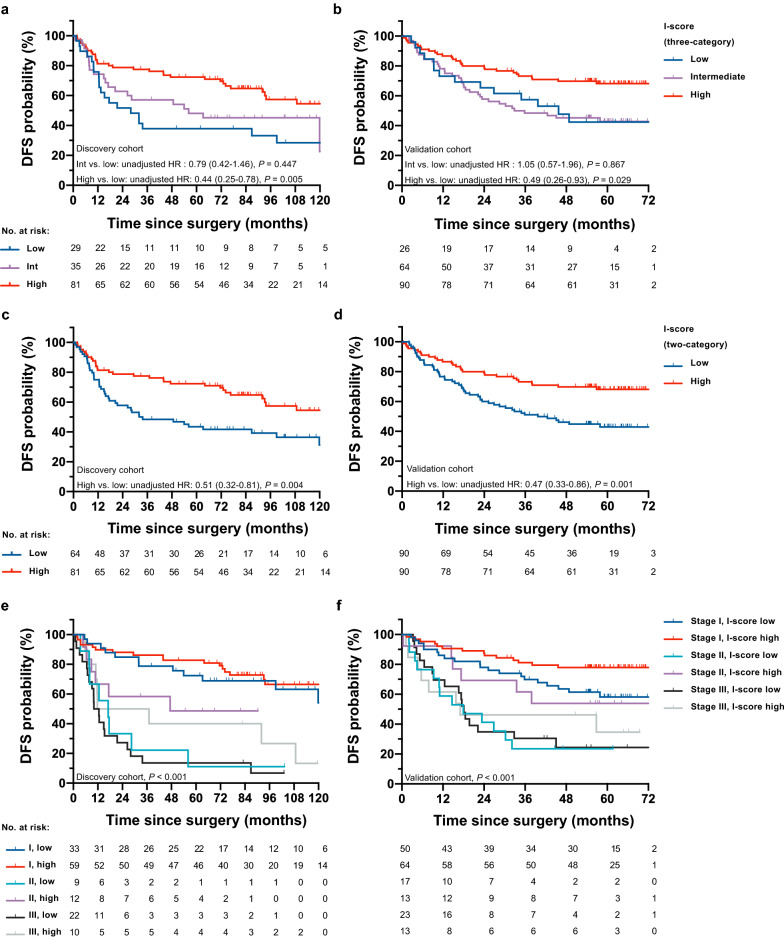
Kaplan–Meier curves of patients stratified by three-category I-score, two-category I-score, and two-category I-score and TNM stage. Compared with a low I-score (three-category), a high I-score is associated with superior DFS in discovery cohort (**a** *P* = 0.005) and validation cohort (**b** *P* = 0.029), whereas an intermediate I-score is not significantly associated with DFS in both cohorts (*P* > 0.050). Compared with a low I-score (two-category), a high I-score is associated with significantly superior DFS, in both discovery cohort (**c** *P* = 0.004) and validation cohort (**d** *P* = 0.001). The two-category I-score and TNM stage are significantly associated with DFS in both discovery cohort (**e** *P* < 0.001) and validation cohort (**f** *P* < 0.001). The unadjusted HRs, corresponding 95% confidence intervals, and *P*-values are determined by univariable Cox regression models. *DFS* disease-free survival. *HR* hazard ratio. Data in parentheses are 95% confidence intervals

Using maximally selected rank statistics method, the cutoffs of CD3-score and CD8-score were determined to be 22.82 and 5.13, respectively, (Fig. 2d, Additional file 1: Figure S2 and Figure S3). We developed a three-category and a two-category immune scoring system “I-score” by integrating the classifications of CD3-score and CD8-score based the discovery dataset (Fig. 2e). For the three-category I-score, the number of patients was 29 (20.0%) for I-score-low, 35 (24.1%) for intermediate, and 81 (55.9%) for high in discovery cohort (5-year DFS: 37.9%, 48.1%, and 72.4%); 26 (14.4%) for I-score-low, 64 (35.6%) for intermediate, and 90 (50.0%) for high in validation cohort (5-year DFS: 42.4%, 42.8%, and 68.2%). Kaplan–Meier curves showed that DFS was superior for I-score-high group compared with I-score-low group (discovery cohort, unadjusted hazard ratio [HR], 0.44; 95% CI, 0.25–0.78; *P* = 0.005; Fig. 4a; validation cohort, 0.49; 0.26–0.93; 0.029; Fig. 4b), but no significant difference of DFS was found between I-score-intermediate and I-score-low groups in both cohorts (*P* > 0.050).

**Fig. 5:**
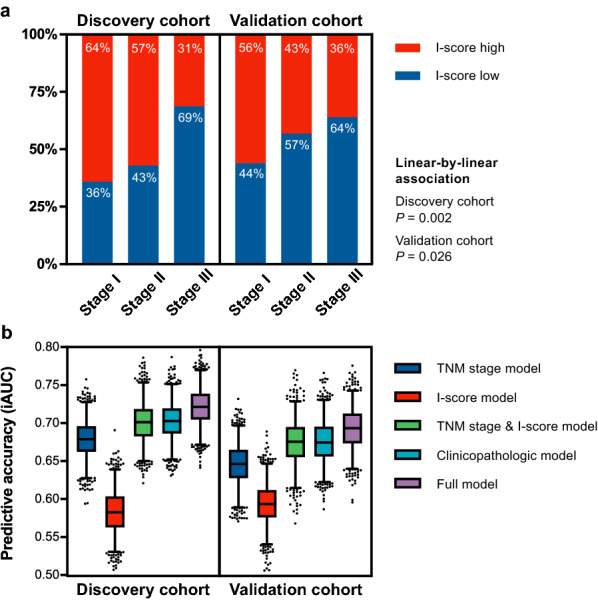
I-score (two-category) distribution across TNM stages and the predictive accuracy of each model in the two cohorts. **a** A low I-score is associated with advanced TNM stages in both discovery cohort (*χ*^2^ = 9.74, *P* = 0.002) and validation cohort (*χ*^2^ = 4.93, *P* = 0.026). **b** The iAUC (resampling with 1000 times bootstrapping) for each model is shown as a box-and-whisker plot; median (lines), interquartile range (boxes), 2.5–97.5 percentile (whiskers). *iAUC* integrated area under the curve

In addition, we constructed a two-category I-score by combining the I-score-low and I-score-intermediate groups in the three-category scoring system. For the two-category I-score, the number of patients were 64 (44.1%) for I-score-low, and 81 (55.9%) for high in discovery cohort (5-year DFS: 43.5%, 72.4%); 90 (50.0%) for I-score-low, and 90 (50.0%) for high in validation cohort (5-year DFS: 43.0%, 68.2%). Kaplan–Meier curves showed that DFS was superior for I-score-high group compared with I-score-low group (discovery cohort, unadjusted HR, 0.51; 95% CI, 0.32–0.81; *P* = 0.004; Fig. 4c; validation cohort, 0.47; 0.33–0.86; 0.001; Fig. 4d). The two-category I-score and TNM stage were associated with DFS in both cohorts (*P* < 0.001; Fig. 4e, Fig. 4f). Besides, we noted that a low I-score was significantly associated with the advanced TNM stage, and this trend could be found in both cohorts (discovery cohort, *χ*^2^ = 9.74, *P* = 0.002; validation cohort, *χ*^2^ = 4.93, *P* = 0.026, Fig. 5a).

Subgroup analyses were further performed, with two cohorts pooling together to increase the discovery power (Additional file 1: Fig. 4). A high I-score (two-category) was associated with significantly superior DFS when stratified by TNM stage (stage I and II), histologic type, differentiation grade, type of surgery (lobectomy/ pneumonectomy), adjuvant chemotherapy, age (< 65 years), sex and smoking history (*P* < 0.050). A similar trend was found in the stage III disease subgroup (Additional file 1: Figure S4c), limited resection subgroup (Additional file 1: Fig. S4i) and 65 years or older subgroup (Additional file 1: Figure S4m), but without statistical significance (*P* > 0.050).

The uni- and multivariable Cox regression analyses for DFS in the two cohorts were presented in Table 2. The factors that reached statistical significance at *P* < 0.10 in the univariable analysis (sex, TNM stage, differentiation grade, adjuvant chemotherapy, two-category I-score) were included in the multivariable analysis. Multivariable analyses demonstrated that I-score (two-category) was independently associated with DFS after adjusting for other clinicopathologic factors (discovery cohort, adjusted HR, 0.54; 95% CI, 0.33–0.86; *P* = 0.010; validation cohort, 0.57, 0.36–0.92; *P* = 0.022).

**Table 2:** Uni- and multivariable Cox regression analyses for DFS in the discovery cohort and validation cohort

Variables	Discovery cohort					Validation cohort			
	Univariable analysis		Multivariable analysis^a^			Univariable analysis		Multivariable analysis^a^	
	Unadjusted HR^b^ (95% CI)	*P*	Adjusted HR^c^ (95% CI)	*P*		Unadjusted HR^b^ (95% CI)	*P*	Adjusted HR^c^ (95% CI)	*P*
Age at surgery (years)									
≥ 65 vs. < 65	1.13 (0.70–1.82)	0.624				1.00 (0.58–1.73)	0.991		
Sex									
Female vs. male	0.63 (0.39–1.03)	0.067				0.58 (0.36–0.94)	0.026		
Smoking status									
Former/current vs. never	1.08 (0.66–1.78)	0.748				1.18 (0.76–1.84)	0.469		
TNM stage									
Stage II vs. stage I	3.82 (1.99–7.34)	< 0.001	2.41 (1.13–5.18)	0.024		2.95 (1.68–5.18)	< 0.001	2.87 (1.64–5.06)	< 0.001
Stage III vs. stage I	5.69 (3.33–9.73)	< 0.001	2.84 (1.33–6.06)	0.007		3.62 (2.15–6.09)	< 0.001	3.23 (1.91–5.48)	< 0.001
Differentiation grade									
G3/G4 vs. G1/G2	2.79 (1.72–4.52)	< 0.001	1.68 (1.01–2.82)	0.047		1.56 (1.00–2.43)	0.050		
Surgical resection									
Limited resection vs. lobectomy/pneumonectomy	1.37 (0.63–2.99)	0.429				1.56 (0.49–4.94)	0.451		
Adjuvant chemotherapy									
Yes vs. no	3.83 (2.38–6.15)	< 0.001	1.77 (0.91–3.41)	0.090		2.23 (1.35–3.68)	0.002		
I-score (three-category)									
Intermediate vs. low	0.79 (0.42–1.46)	0.447				1.05 (0.57–1.96)	0.867		
High vs. low	0.44 (0.25–0.78)	0.005				0.49 (0.26–0.93)	0.029		
I-score (two-category)									
High vs. low	0.51 (0.32–0.81)	0.004	0.57 (0.36–0.92)	0.022		0.47 (0.30–0.75)	0.001	0.54 (0.33–0.86)	0.010

Data in parentheses are 95% confidence intervals

*DFS*  disease-free survival, *HR* hazard ratio, *CI*  confidence interval

^a^Variables that reach statistical significance at *P* < 0.10 in the univariable analysis (sex, TNM stage, differentiation grade, adjuvant chemotherapy, two-category I-score) are included in the multivariable analysis

^b^The unadjusted hazard ratios (HR) and *P*-values are determined by univariable Cox regression analyses

^c^The adjusted hazard ratios (HR) and *P*-values are determined by multivariable Cox regression analyses

### Development and validation of prognostic prediction models

Since I-score (two-category), TMN stage, differentiation grade, and adjuvant chemotherapy were identified as independent factors of DFS in the discovery cohort, we developed a prognostic prediction model (full model) based on the factors above. We further compared the performance of the full model with four other models that included a TNM stage model, I-score model, TNM stage & I-score model, and clinicopathologic model (TMN stage & differentiation grade & adjuvant chemotherapy). The coding, partial regression coefficients and estimated 5-year baseline cumulated hazard of each model were summarized in Additional file 1: Table S1.

The model performance metrics were presented in Table 3. The full model showed better discrimination (evaluated by iAUC and C-index) and calibration (evaluated by AIC) than the clinicopathologic model in both cohorts (discovery cohort, iAUC, 0.717 vs. 0.698; C-index, 0.742 vs. 0.728; AIC, 610.9 vs. 614.2; validation cohort, iAUC, 0.684 vs. 0.671; C-index, 0.695 vs. 0.685; AIC, 734.8 vs. 739.2). The TNM-stage & I-score model showed better discrimination and calibration than the TNM-stage model in both cohorts (discovery cohort, iAUC, 0.699 vs. 0.674; C-index, 0.711 vs. 0.694; AIC, 613.5 vs. 615.6; validation cohort (iAUC, 0.673 vs. 0.645; C-index, 0.679 vs. 0.651; AIC, 736.4 vs. 742.3). ﻿Integrating the I-score into a TNM stage model improved the prediction for DFS (likelihood ratio *P* = 0.044, Fig. 5b); also, integrating the I-score into a clinicopathologic model improved the prediction for DFS (likelihood ratio *P* = 0.022, Fig. 5b).

**Table 3:** Performance metrics for integrated I-score (two-category) models and reference models

Models	Discovery cohort				Validation cohort		
	iAUC^a^	Harrell’s C-index	AIC		iAUC	Harrell’s C-index	AIC
TNM stage model ^b^	0.674	0.694 (0.640–0.749)	615.6		0.645	0.651 (0.596–0.705)	742.3
I-score model	0.584	0.592 (0.532–0.651)	647.4		0.592	0.588 (0.533–0.644)	758.6
TNM Stage & I-score model ^b^	0.699	0.711 (0.651–0.772)	613.5		0.673	0.679 (0.623–0.736)	736.4
Clinicopathologic model ^c^	0.698	0.728 (0.676–0.781)	614.2		0.671	0.685 (0.627–0.743)	739.2
Full model ^c^	0.717	0.742 (0.688–0.795)	610.9		0.684	0.695 (0.639–0.751)	734.8

Data in parentheses are 95% confidence intervals

*iAUC* integrated area under the curve, *Harrell’s C-index* Harrell’s concordance index, *AIC* Akaike information criterion

^a^iAUC refers to the integrated area under the ROC curve

^b^ TNM-stage model vs. TNM Stage & I-score model: likelihood ratio *P* = 0.044

^c^Clinicopathologic model (TMN stage & differentiation grade & adjuvant chemotherapy) vs. Full model: likelihood ratio *P* = 0.022

## Discussion

In this study, we developed an automated workflow for evaluating the density of CD3^+^ and CD8^+^ cells in the tumour regions on IHC-stained WSIs of NSCLC, and further proposed an immune scoring system “I-score” based on the automated assessed cell density. The generalizability of this automated workflow and novel scoring system was validated in an external independent cohort. To the best of our knowledge, this is the first study that utilized automated whole-slide images assessment of tumour-infiltrating CD3^+^ and CD8^+^ T-cells for the prognostic stratification of resected NSCLC.

The past 10 years have seen remarkable progress in medical artificial intelligence, promoting the development of digital pathology. Digital pathology implies not only the digitization of tissue sections, but also the automated assessment workflow with high validity and reliability. The application of WSIs has expanded the scope of histopathological analyses to a whole-slide level, which places higher demands on automated algorithms. Some earlier pioneering WSI-based studies predicted the prognosis of NSCLC based on automated derived image features (e.g. Haralick texture features, radial distribution of pixel intensity, etc.) [27], or predicted the classification and mutation status using end-to-end deep learning models in a data-driven manner [28], which had limitations in ﻿biological interpretability.

Analysing the tumour microenvironment at the tissue and cellular levels depends on precise segmentation and identification methods, but the high histologic heterogeneity in NSCLC presents a challenge to algorithm development [29]. This study optimized the automated positive cells assessment algorithm in the following two aspects. In the tissue segmentation process, we used a semi-automated interactive approach combining the automated algorithm and the experience of pathologists. The tumour region was determined by precisely removing adjacent normal tissues, blanks, and backgrounds to reduce the errors in estimating the tumour area. The tumour-adjacent atelectasis (belongs to normal tissue) was easily confused with tumour-associated stroma (belongs to tumour region) in this thresholding segmentation framework, so the experience of the pathologist was dispensable for identifying these tissues. The blank area (residual alveolar cavity) was a unique structure for lung cancer tissue sections, and its size varied with histologic subtypes [30]. In previous studies, the density of positive cells was defined as the counting of positive cells per unit area (mm^2^) [31], and the area could be the high power field [32] or the tissue surface area [14]. Some other studies defined the density as the percentage of positive cells among total nucleated cells [14, 33]. Our study calculated the density of positive cells using tissue surface area as the denominator, and the evaluation would be robust across histologic subtypes. As a result, the I-score based on the density of CD3^+^ and CD8^+^ T-cells showed good stratification performance in the adenocarcinoma and squamous cell carcinoma subgroup (Additional file 1: Figure S4d, e). In the cell segmentation process, dust macules (similar to, but slightly darker than positive cells) were filtered out to avoid being mistakenly identified as positive cells. As a result, there was a good agreement between manual counting and automated counting using our algorithm (ICC, 0.91).

Although for colon cancer, there has been a well-developed workflow for WSI assessment of Immunoscore [18], a generally accepted immune scoring system for NSCLC prognostic stratification is still unavailable. Selecting which types of ﻿TILs and which regions/compartments of ﻿TILs for scoring has always been controversial. We referred to the findings of previous Immunoscore-related studies on NSCLC [13, 14], and selected CD3^+^ (pan T-cells) and CD8^+^ (cytotoxic T-cells), two robust prognosis-associated markers in various solid cancers including NSCLC [10, 31], for quantitative assessments. Concerning the regions for cell quantification, some studies (especially TMA-based studies) quantified the positive cells in the central tumour and the invasive margins respectively [33, 34]. Instead, we constructed the immune scoring system based on the positive cell density in the entire tumour regions (tumour nests) on WSIs, as in some previous studies [14, 35]. Therefore, the characteristics of immune infiltrations in the central tumour and the invasive margins (if it existed on a WSI) had been taken into account, and the selection bias could be reduced.

The I-score (two-category) that integrated the CD3-score and the CD8-score was associated with DFS after adjusting for TNM stage and other clinicopathologic factors. This finding was verified in an external validation cohort with significant differences in baseline characteristics compared with discovery cohort, suggesting that the I-score obtained by the automated workflow was an independent and robust prognostic factor of DFS in resected NSCLC. Furthermore, the prognostic value of the I-score was confirmed in the vast majority of subgroups (Additional file 1: Figure S4). The predictive accuracy (iAUC and C-index, C-index: 0.588 vs. 0.58 for validation cohort) of the I-score was similar to that of the Immunoscore of colon cancer [36]. By integrating the I-score (two-category) into the TNM stage model and clinicopathologic model, respectively, the models with I-score showed better discrimination and calibration than those without I-score in both cohorts (Fig. 5b), which suggested that the I-score based on the automated assessed cell density would improve the prognostic risk stratification in resected NSCLC. Also, the full model yielded better discrimination compared with the reported prediction models that involved only clinicopathologic prognostic factors [6–8] (C-index, 0.695 vs. 0.67, 0.664, 0.66 for validation cohort).

As for the I-score distribution across TNM stages, an interesting trend was found that a low I-score was significantly associated with the advanced TNM stage. We speculated that this might be attributable to the evolution of immune escape. A similar finding was reported in a recent genomic study on the spectrum of immune infiltration from preneoplasia to invasive lung adenocarcinomas [37]. Still, the underlying mechanism of these findings warrants further investigation.

This study has limitations inherent to most retrospective studies. The clinical validity of this automated workflow and immune scoring system needs to be further validated in larger prospective cohorts. Besides, the quality control of WSIs was performed manually, and some parameters for tumour region segmentation were fine-tuned, if required, according to the pathologists’ proofreading. Based on the findings in this study, we are currently developing a deep-learning framework to perform NSCLC tissue segmentation, which would enable automated segmentation and identification of tumour regions and tumour-associated stroma. The density of CD8^+^ cells in the stroma compartment was reported to be an independent prognostic factor in resected NSCLC [10, 33, 38], and a precise segmentation algorithm would be an essential prerequisite for evaluating immune infiltration in the stroma compartment.

## Conclusion

In summary, we presented an automated workflow for characterizing the immune infiltration in the entire tumour regions based on IHC-stained WSIs, and proposed an immune scoring system “I-score” based on the automated assessed cell density. This automated workflow and novel scoring system would advance the clinical application of immune microenvironment evaluation with satisfactory validity and reliability. This study suggested that integration of I-score into clinicopathological risk factors would improve the prognostic stratification, and support the clinical decision making for patients with resected NSCLC.

## Supplementary Information

**Additional file 1: Note S1.** Immunohistochemical staining. **Table S1.** The coding, partial regression coefficient and estimated 5-year baseline cumulative hazard of each prediction model. **Figure S1.** Comparison of automated and manual counting of positive cells. **Figure S2.** Determination of an optimal cut-off for CD3-score. **Figure S3.** Determination of an optimal cut-off for CD8-score. **Figure S4.** Kaplan-Meier curves of subgroup analyses

## Data Availability

The tile-level images and the code for the automated algorithms that used in this study are available from the corresponding authors upon reasonable request.

## Abbreviations

NSCLC: Non-small-cell lung cancer
IHC: Immunohistochemistry
WSI: Whole-slide image
DFS: Disease-free survival
HR: Hazard ratio
CI: Confidence interval
TNM: Tumour-node-metastasis
TIL: Tumour-infiltrating lymphocyte
TMA: Tissue microarray
ICC: Intraclass correlation coefficient
AIC: Akaike information criterion
iAUC: Integrated area under the curve
C-index: Harrell’s concordance index
