# Supplementary material for: Automated whole-slide images assessment of immune infiltration in resected non-small-cell lung cancer: towards better risk-stratification
Source: J Transl Med. 2022 Jun 7;20:261. doi: 10.1186/s12967-022-03458-9 (PMC9172185; doi:10.1186/s12967-022-03458-9)
Supplement: Supplementary file 1 — Additional file 1: Note S1. Immunohistochemical staining. Table S1. The coding, partial regression coefficient and estimated 5-year baseline cumulative hazard of each prediction model. Figure S1. Comparison of automated and manual counting of positive cells. Figure S2. Determination of an optimal cut-off for CD3-score. Figure S3. Determination of an optimal cut-off for CD8-score. Figure S4. Kaplan-Meier curves of subgroup analyses [file 12967_2022_3458_MOESM1_ESM.docx]

**Additional file 1 for**

**“Automated whole-slide images assessment of immune infiltration in resected non-small-cell lung cancer: towards better risk-stratification”**

**Contents:**

1. Note S1. Immunohistochemical staining
2. Table S1. The coding, partial regression coefficient and estimated 5-year baseline cumulative hazard of each prediction model
3. Figure S1. Comparison of automated and manual counting of positive cells.
4. Figure S2. Determination of an optimal cut-off for CD3-score
5. Figure S3. Determination of an optimal cut-off for CD8-score
6. Figure S4. Kaplan-Meier curves of subgroup analyses

**Additional file Note S1. Immunohistochemical staining**

The immunohistochemistry staining was performed on an automated immunostainer. After deparaffinization, microwave antigen retrieval was performed in citrate buffer pH6. Nonspecific binding was blocked using 10% normal goat serum (Tian Gen, China). The slides were incubated at 4 ℃ overnight with anti-CD3 mAb (05278422001, Ventana) and anti-CD8 mAb (GM710329, Gene Tech). Horseradish peroxidase-conjugated secondary antibodies (Santa Cruz Biotechnology, Dallas, TX) were used for visualization. All tissue sections were counterstained with haematoxylin.

**Additional file Table S1. The coding, partial regression coefficient and estimated 5-year baseline cumulative hazard of each prediction model**

| **Models** | **Baseline** **hazard** | **Factors** | **Coding** | **Regression coefficients** |
| --- | --- | --- | --- | --- |
| TNM stage model | 0.503 | Stage II (dummy variable) | Stage I=0, Stage II=1, Stage III=0 | 1.340 |
|  |  | Stage III (dummy variable) | Stage I=0, Stage II=0, Stage III=1 | 1.740 |
| I-score model | 0.514 | I-score (two-category) | Low=0, High=1 | -0.682 |
| TNM stage &  I-score model | 0.498 | Stage II (dummy variable) | Stage I=0, Stage II=1, Stage III=0 | 1.339 |
|  |  | Stage III (dummy variable) | Stage I=0, Stage II=0, Stage III=1 | 1.649 |
|  |  | I-score (two-category) | Low=0, High=1 | -0.488 |
| Clinicopathologic model | 0.483 | Stage II (dummy variable) | Stage I=0, Stage II=1, Stage III=0 | 0.927 |
|  |  | Stage III (dummy variable) | Stage I=0, Stage II=0, Stage III=1 | 1.171 |
|  |  | Differentiation grade | G1/G2=0, G3/G4 =1 | 0.470 |
|  |  | Adjuvant chemotherapy | No=0, Yes=1 | 0.513 |
| Full model | 0.480 | Stage II (dummy variable) | Stage I=0, Stage II=1, Stage III=0 | 0.881 |
|  |  | Stage III (dummy variable) | Stage I=0, Stage II=0, Stage III=1 | 1.044 |
|  |  | Differentiation grade | G1/G2=0, G3/G4 =1 | 0.522 |
|  |  | Adjuvant chemotherapy | No=0, Yes=1 | 0.569 |
|  |  | I-score (two-category) | Low=0, High=1 | -0.557 |

**Additional file Figure S1. ﻿Comparison of automated and manual counting of positive cells.**

The result of positive cell segmentation using our algorithm (**a**); QuPath built-in algorithm, minimum cell area threshold at 100 pixel^2^ (**c**); QuPath built-in algorithm, threshold at 150 pixel^2^ (**e**); QuPath built-in algorithm, threshold at 200 pixel^2^ (**g**). Compared to the QuPath built-in algorithm, our algorithm shows better segmentation performance, with less over-segmentation (yellow arrows) and under-segmentation (white arrows). The Bland-Altman plots show good agreement between the manual counting and automated counting using our algorithm (intraclass correlation coefficient [ICC], 0.91; 95% confidence interval [CI], 0.87–0.94; *P*<0.001; **b**), and show moderate agreement between the manual counting and automated counting using QuPath built-in algorithm (minimum cell area threshold at 100 pixel^2^, ICC, 0.72; 95% CI, 0.21–0.88; *P*<0.001; **d**; threshold at 150 pixel^2^, ICC, 0.72; 95% CI, 0.36–0.86; *P*<0.001; **f**; threshold at 200 pixel^2^, ICC, 0.91; 95% CI, -0.09–0.73; *P*<0.001; **h**). ICC=intraclass correlation coefficient. Data in parentheses are 95% confidence intervals.

**Additional file Figure S2. ﻿Determination of an optimal cut-off for CD3-score.**

**a.** The distribution of CD3-score in the discovery cohort. **b.** The optimal cut-off of the CD3-score is determined by the maximally selected rank statistics method. Those with a CD3-score higher than 22.82 are defined as CD3-high, otherwise as CD3-low.

**Additional file Figure S3. Determination of an optimal cut-off for CD8-score.**

**a.** The distribution of CD8-score in the discovery cohort. **b.** The optimal cut-off of the CD8-score is determined by the maximally selected rank statistics method. Those with a CD8-score higher than 5.13 are defined as CD8-high, otherwise as CD8-low.

**Additional file Figure S4. Kaplan-Meier curves of subgroup analyses.**

Kaplan-Meier curves of patients stratified by two-category I-score in the subgroup of patients with stage I disease (**a**), stage II disease (**b**), stage III disease (**c**), adenocarcinoma (**d**), squamous cell carcinoma (**e**), well-moderately differentiated (G1/G2) cancer (**f**), poorly-undifferentiated (G3/G4) cancer (**g**), lobectomy/ pneumonectomy (**h**), limited resection (**i**), adjuvant chemotherapy (**j**), without adjuvant chemotherapy (**k**), age younger than 65 years (**l**), 65 years or older (**m**), male sex (**n**), female sex (**o**), never smoking history (**p**), former/current smoking history (**q**). Except for stage III disease subgroup (**c**), limited resection subgroup (**i**) and 65 years or older subgroup (**m**), a high I-score (two-category) is associated with significantly superior DFS in the pooled dataset (the solid lines, whereas the dash lines indicates discovery cohort and validation cohort). DFS=disease-free survival. HR=hazard ratio. Data in parentheses are 95% confidence intervals.
